# Neurogenomic Evidence for a Shared Mechanism of the Antidepressant Effects of Exercise and Chronic Fluoxetine in Mice

**DOI:** 10.1371/journal.pone.0035901

**Published:** 2012-04-25

**Authors:** Guo-Jen Huang, Eyal Ben-David, Agnès Tort Piella, Andrew Edwards, Jonathan Flint, Sagiv Shifman

**Affiliations:** 1 Department and Graduate Institute of Biomedical Sciences, College of Medicine, Chang Gung University, Tao-Yuan, Taiwan; 2 Department of Genetics, The Institute of Life Sciences, The Hebrew University of Jerusalem, Jerusalem, Israel; 3 Wellcome Trust Centre for Human Genetics, University of Oxford, Oxford, United Kingdom; Mayo Clinic College of Medicine, United States of America

## Abstract

Several different interventions improve depressed mood, including medication and environmental factors such as regular physical exercise. The molecular pathways underlying these effects are still not fully understood. In this study, we sought to identify shared mechanisms underlying antidepressant interventions. We studied three groups of mice: mice treated with a widely used antidepressant drug – fluoxetine, mice engaged in voluntary exercise, and mice living in an enriched environment. The hippocampi of treated mice were investigated at the molecular and cellular levels. Mice treated with fluoxetine and mice who exercised daily showed, not only similar antidepressant behavior, but also similar changes in gene expression and hippocampal neurons. These changes were not observed in mice with environmental enrichment. An increase in neurogenesis and dendritic spine density was observed following four weeks of fluoxetine treatment and voluntary exercise. A weighted gene co-expression network analysis revealed four different modules of co-expressed genes that were correlated with the antidepressant effect. This network analysis enabled us to identify genes involved in the molecular pathways underlying the effects of fluoxetine and exercise. The existence of both neuronal and gene expression changes common to antidepressant drug and exercise suggests a shared mechanism underlying their effect. Further studies of these findings may be used to uncover the molecular mechanisms of depression, and to identify new avenues of therapy.

## Introduction

A diverse set of interventions, in addition to drugs, is known to have antidepressant action, including cognitive and electro-convulsive therapies, sleep deprivation, and exercise [1], [2]. Mechanistic understanding of what is common to environmental and drug treatments may provide valuable clues about the mode and site of action of antidepressants. Recent studies demonstrate the importance of adult hippocampal neurogenesis for the action of antidepressants [3], [4]. Both exercise and enriched environment have also been found to increase hippocampal neurogenesis [5] and cause antidepressant-like behavioral change. Chronic exercise reduces depressive-like behavior in rats [6], [7] and mice [8], as measured in standard models of depression such as the forced swim test. Environmental enrichment has similar consequences [9] and both produce notably similar effects on the brain in stimulating cell proliferation and recruitment of new neurons into the dentate gyrus of the hippocampus [5], [10], [11].

Currently no molecular pathway is known that is common to these treatments. The finding that gene expression data show structured correlation, together with the development of weighted gene co-expression network analysis (WGCNA) [12], [13], provide a system-level approach for using gene expression to detect the common mechanisms of different interventions. WGCNA organizes genes into modules that are co-regulated and therefore are more likely to be functionally related and to participate in similar cellular processes. WGCNA also alleviates the multiple testing problem inherent in testing tens of thousands of transcripts, a problem that otherwise substantially reduces the power of standard differential expression analysis. Instead of testing the changes in expression of each of thousands of genes, a small number of gene co-expression modules are tested in the WGCNA approach.

In this study we look for common molecular and neuronal mechanisms for antidepressant action by studying the hippocampi of mice exposed to three different interventions. We compared changes in adult neurogenesis, neuronal plasticity, and gene expression induced by exercise, environmental enrichment, and fluoxetine, a specific serotonin reuptake inhibitor commonly prescribed for major depression. We examined these phenotypes in the hippocampus, because this brain structure has been implicated in the pathophysiology and treatment of mood disorders, and changes in adult neurogenesis in the hippocampus are associated with exercise, environmental enrichment and possibly also with the therapeutic effect of antidepressants.

## Materials and Methods

### Ethics statement

This study was carried out in strict accordance with the recommendations in the Guide for Laboratory Animals Facilities and Care as promulgated by the Council of Agriculture. Executive Yuan, ROC. The protocol was approved by the Institutional Animal Care and Use Committee of Chang Gung University (Permit Number: CGU11-008). In this Protocol, all efforts were made to minimize suffering.

### Animals

Eight week old C57BL/6J male mice were individually housed in standard cages (28×35×13 cm) with ad libitum access to food and water for 28 days. Groups of mice were treated with one of the following four protocols: (1) Exposure to an enriched environment: cages were filled with toys, including tubes, ladders, balls and shelters (but not a running wheel). Toys were changed every three days. (2) Voluntary exercise: mice were housed with a free access to a running wheel (Bio-Serv, US). Running distance on the wheel was measured using standard bicycle odometers (Cateye Velo 8). Previous studies showed that mice given access to running wheels for 3–4 weeks showed robust antidepressant behavior [8] (3) Chronic fluoxetine treatment: The mice were given fluoxetine (80 mg/L) in their drinking water. Average intake was 16 mg/kg/day of fluoxetine. Administration of this dose by oral administration for 28 days was shown before to produce a robust antidepressant behavior in mice and increase in neurogenesis in the hippocampus [3] (4) The control group: the control group of mice was not exposed to any of the treatments. All groups were housed at a controlled environment and kept on a 12 hour light/dark cycle.

### Immunohistochemistry

All sections for KI67 and DCX staining were cut to a thickness of 40 µm on a sliding microtome. For KI67 staining, sections were mounted on the superfrost slides (BDH, UK) and dried overnight. Subsequently, slides were incubated in the 0.01 mol/L citric buffer for 40 min at 90°C, 3% H_2_O_2_ for 10 min, rinsed in PBS, and incubated overnight at room temperature in rabbit anti-KI67 antibody (1∶4000, Vector Lab). Next day, a standard rabbit IgG ABC kit (Vector Lab) procedure was used and the slides reacted for 5–10 min with Sigma DAB tablet. Sections were then counterstained with cresyl violet and cover-slipped with DPX. KI67-labeled cells were counted bilaterally on every eighth section through the entire rostrocaudal extent of the granule cell layer. For DCX staining, free floating sections were incubated with goat anti-DCX antibody (1∶400, Santa Cruz Labs), then following the same ABC kit procedure, reacted with Sigma DAB tablet. DCX-labeled cells were counted bilaterally on every sixteenth section through the entire rostrocaudal extent of the granule cell layer.

### Fluorescent staining

For BrdU/NeuN double labeling, fluorescent staining was performed on 40 µm floating sections. The sections were incubated in 2M HCl for 30 min at 37°C, neutralised in boric acid (Sigma) for 15 min (pH 8.5) and washed 3 times in PBS before incubation with BrdU antibodies (Accurate Chemical and Scientific; 1∶ 200) and NeuN (Chemicon; 1∶ 400). Following three washes in PBS (5 min each), sections were incubated with the fluorescent secondary antibody (1∶ 200, Alex Fluor 488 and Alex Fluor 568, Invitrogen) for 2 h in 0.3% Triton/PBS with 2% of goat serum. Images of sections were captured on a Zeiss LSM 510 META confocal microscope.

### Golgi staining

Brains were harvested and stained using an FD rapid Golgistain™ Kit in accordance with the manufacturer's instruction (FD Neuro Technologies) (n = 3 for control group, n = 4 for exercise, enriched and fluoxetine groups). Brains were sectioned coronally at a thickness of 80 µm using a microtome. Only spines from apical CA1 dendrites in matched mid hippocampus sections were used for analysis. Ten dendrites per brain were photographed using an Olympus BX-51 microscope equipped with a 100× objective. Images freeware was used to measure dendrite length and manually count spine number.

### Expression analysis

The hippocampi of mice were isolated after 28 days and were immediately frozen on dry ice. The hippocampi were homogenized using 5-mm stainless steel beads (Qiagen) on a Tissue Lyser (Retsch MM300 Mixer Mill) for 10 min at 25 Hz. Total RNA was extracted using the RNeasy Lipid Tissue Kit (Qiagen) according to the manufacturer's instructions. RNA quantity and integrity were assessed using a NanoDrop ND-1000 Spectrophotometer and an Agilent 2100 Bioanalyser. All RNA samples had an RNA Integrity Number (RIN)>9.

One microgram of total RNA was used as starting material for the expression analysis. The ribosomal RNA was removed using a RiboMinus Mouse Transcriptome Isolation Kit (Invitrogen). RNA samples were further processed according to the manufacturer's protocol using Affymetrix's GeneChip Whole Transcript Sense Target Labeling Assay. Fragmented and biotinylated cDNA were added to the hybridization mixture and loaded on a Affymetrix GeneChip Mouse Exon 1.0 ST array. After hybridization, the array was washed and stained according to Affymetrix protocol using the GeneChip Fluidics Station 450/250. The stained array was scanned using an Affymetrix GeneChip Scanner 3000. Quality control was carried out using Affymetrix Expression Console Software Version 1.0. Signal estimates were derived from the CEL files by quantile sketch normalization using the IterPLIER for gene-level intensities using the Expression Console software (Affymetrix). Only “core” level probe sets (probe sets assigned to the highest confidence level) were used in the analysis. Gene-level iterPLIER estimates are derived by combining correlated probe sets, predicted to map into the same transcript cluster (according to the meta-probe set list). The iterPLIER algorithm iteratively discards probes that do not correlate well with the overall gene-level signal and then recalculates the signal estimate to derive a robust estimation of the gene expression value. Genes were considered as expressed based on a “Detection Above Background” (DABG) *P*<0.05 for 80% or more of the samples. Processed data were exported to the R statistical computing language and analyzed using analysis of variance (ANOVA). The q-value (the proportion of false positives), for false discovery rate control, was estimated using the ‘qvalue’ package for R. Differential transcripts were identified as those with q-value≤0.05. The cell files from the study of Miller et al. that examine the effect of fluoxetine on gene expression were retrieved from the Gene Expression Omnibus (GEO), and reanalyzed using Affymetrix Expression Console and normalized using the RMA algorithm. The data and the experimental details can be found in the GEO website (http://www.ncbi.nlm.nih.gov/geo/query/acc.cgi?acc=GSE6476). In addition, we examined unpublished data that examined the effect of chronic treatment of Clozapine and Haloperidol on gene expression in the mouse brain (http://www.ncbi.nlm.nih.gov/geo/query/acc.cgi?acc=GSE6511).

### Network analysis

A weighted gene coexpression network was constructed according to previously described methodology. The network was constructed using the 4000 most variable genes across all samples. The network was constructed based on a pairwise correlation matrix of the expression values. To approximate a scale-free network topology, the data was transformed by a fixed power (β = 14). To identify gene modules, we followed the standard WGCNA approach. Hierarchical clustering was performed on the topological overlap dissimilarity matrix and the tree was cut using a dynamic tree-cutting algorithm. Modules were merged together if the modules eigengenes were highly correlated (a correlation above 0.8). To test for association between antidepressants and gene modules, the eigengene of each module was tested for significant correlation to the trait. Testing for overrepresentation of functional categories was carried out using Database for Annotation, Visualization, and Integrated Discovery (DAVID) tools, version 6.7 [14], [15]. Categories analyzed included GO categories, pathways database (KEGG Pathways), and functional categories (SP PIR Keywords). The Benjamini correction for multiple testing was applied with a threshold for an adjusted *P*<0.05.

### Behavior

For the open field test, mice were placed in a brightly lit, white, and circular arena with a 60 cm diameter. This was divided into an inner (40 cm in diameter) and an outer circular area. Mouse movements were monitored for 5 min via an automated tracking system (Videotrack. vNT4.0: Viewpoint). Marble burying was tested by placing the mice individually into plastic cages (20×30 cm) containing a 5 cm layer of sawdust bedding and 12 glass marbles. Cages were placed on a Threshold system (Med Associates Inc., St. Albans, VT, USA) to monitor digging/burying behavior for 30 min.

For novelty suppressed feeding, mice were food deprived for 24 h and then placed in a novel rodent cage (30×50 cm) that contained mouse predator odor (rat feces and fur) for 6 min. Four regular food pellets were placed in the corner of the cage. The total time that the mice spent eating was recorded. After the test, mice were placed back in their home cage with food pellets. The consumption of food pellets (g) were measured within the first 30 min. For novelty induced hypophagia, mice were given the sweetened milk in their home cage, twice a day for three days. On the fourth day, the latency and time spent drinking within 6 min in their home cage under dim lighting conditions was measured. On the fifth day, the latency to time spent on the drinking in their home cage after relocation to a novel, brightly lit room was scored.

## Results

### Fluoxetine and voluntary exercise increase neurogenesis and spine density

**Figure 1:**
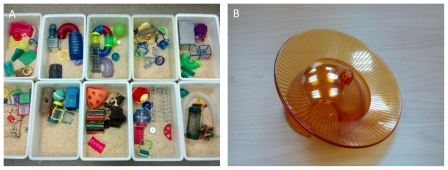
The enriched environment and running wheel used in this study. (A) The photo shows cages with different toys, including tubes, ladders, houses, balls. The toys in the enriched cages were changed every three days. (B) The running wheel used with the exercise group.

C57BL/6J mice (8 weeks old, n = 10) were divided into four groups. All the mice were given one injection of BrdU to label the dividing cells (ip, 200 mg/kg). The first group was exposed to an environment enriched with toys, including different shaped tubes, ladders, balls and housing (Figure 1A), which were changed every three days. A second group of mice was housed with free access to a running wheel (Figure 1B). The average running distance, as measured by standard bicycle odometers, was 12 km per day. The third group was given fluoxetine in their drinking water (80 mg/L). A fourth control group of ten mice was not exposed to any of the above treatments.

**Figure 2:**
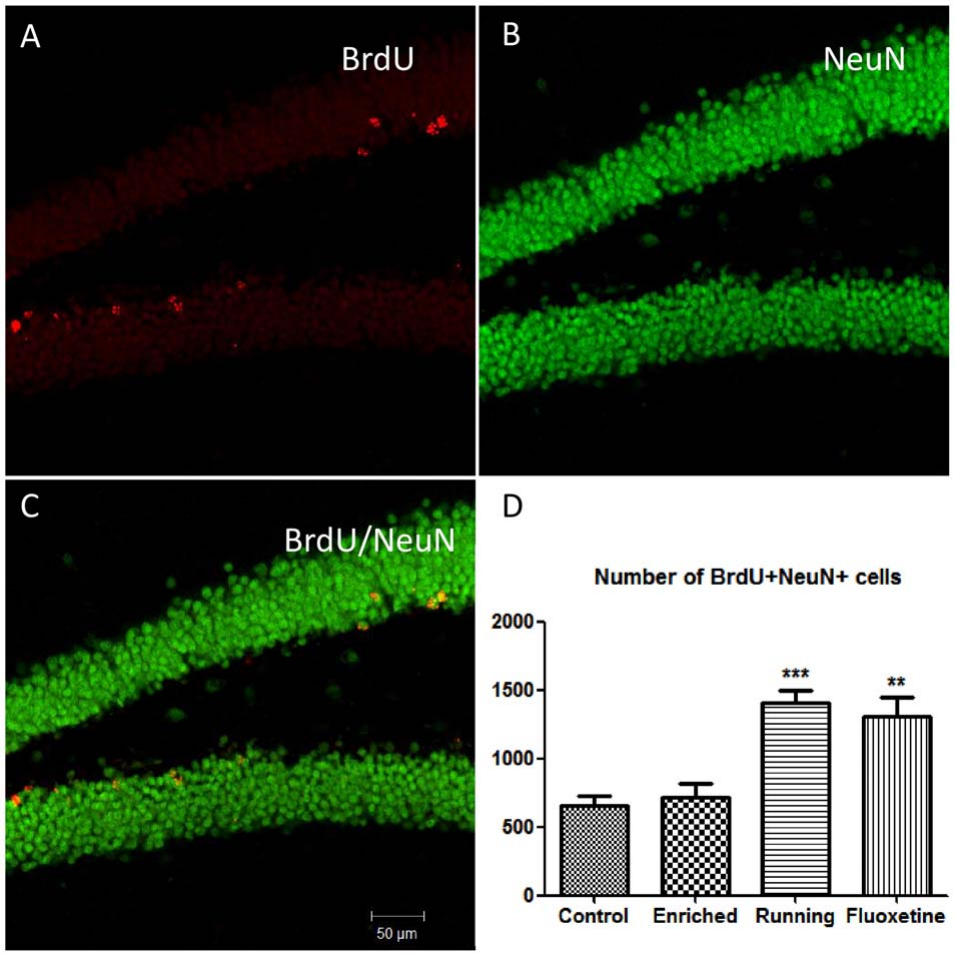
Cell survival and neurogenesis in the four different groups. BrdU (200 mg/kg) was injected into all mice before dividing them into different groups. After 28 days, brain tissues (hippocampus) were stained for BrdU and NeuN (A–C). The exercise and fluoxetine groups, but not the enriched environment group, showed higher number of BrdU and NeuN positive cells in the dentate gyrus (D) compared to the control group. Values are means ± SEM. **P<0.01, ***P<0.001.

**Figure 3:**
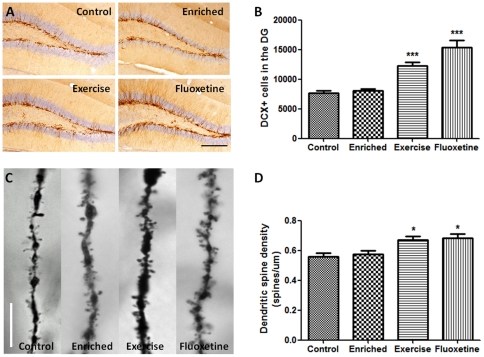
DCX staining and dendritic spine density in the hippocampus. (A and B) The exercise and fluoxetine groups showed more immature neurons in the dentate gyrus compared to controls. (C and D) Golgi staining images reveal higher level of dendritic spine density in the hippocampus (CA1 region) in the exercise and fluoxetine groups. Values are means ± SEM. *P<0.05, ***P<0.001. Scale bar show 200 µm (A) and 10 µm (B).

After 28 days, the mice were analyzed for the levels of adult neurogenesis (n = 6) and neuronal spine density (n = 4). A higher neuronal survival level was observed in mice belonging to the exercise and fluoxetine groups relative to controls, as indicated by the number of BrdU+/NeuN+ labeled cells (Figure 2 A–D) (exercise vs. control: *P*<0.001, t = 5.15; fluoxetine vs. control: *P*<0.01, t = 4.44). Approximately 80% of the BrdU positive cells were also positive for the neuronal marker, NeuN; however there were no differences in the percentage of BrdU/NeuN co-labeling between the four groups (*P* = 0.76). Unlike the BrdU labeling, a significant increase in KI67, which is a marker for proliferation, was observed only for animals treated with fluoxetine (*P*<0.01, t = 4.42). To estimate the level of ongoing neurogenesis, we used a marker for immature neurons – doublecortin (DCX). Both the exercise and fluoxetine groups showed an increase in neurogenesis as indicated by the DCX staining (Figure 3 A and B) (exercise vs. control: *P*<0.001, t = 4.7; fluoxetine vs. control: *P*<0.001, t = 7.9). We also detected a higher level of neuronal spine density, by sampling from the CA1 region, in animals from both exercise and fluoxetine groups (Figure 3 C and D) (exercise vs. control: *P*<0.05; fluoxetine vs. control: *P*<0.01, t = 3.4). Overall, these results show that both voluntary exercise and fluoxetine treatment, but not enriched environment, increase adult neurogenesis and dendritic spine density after 28 days of intervention.

### Similar changes in gene expression with fluoxetine and exercise interventions

We examined gene expression in the hippocampus of seven mice from each group, after 28 days of treatment. We analyzed 23,238 genes, out of which 11,318 genes (48.7%) were found to be expressed. Using analysis of variance (ANOVA), we identified 87 differentially expressed genes with a q-value<0.05 (Table S1). Gene ontology enrichment analyses and functional annotation clustering identified an enrichment of functional groups including: secreted [SP PIR Keywords] (q-value = 2.0×10^−7^), extracellular region [GO:0005576] (q-value = 2.1×10^−7^), glycoprotein [SP PIR Keywords] (q-value = 1.5×10^−6^), extracellular region [GO:0005576] (q-value = 2.1×10^−7^) and ECM-receptor interaction [KEGG PATHWAY] (q-value = 5.4×10^−3^).

**Figure 4:**
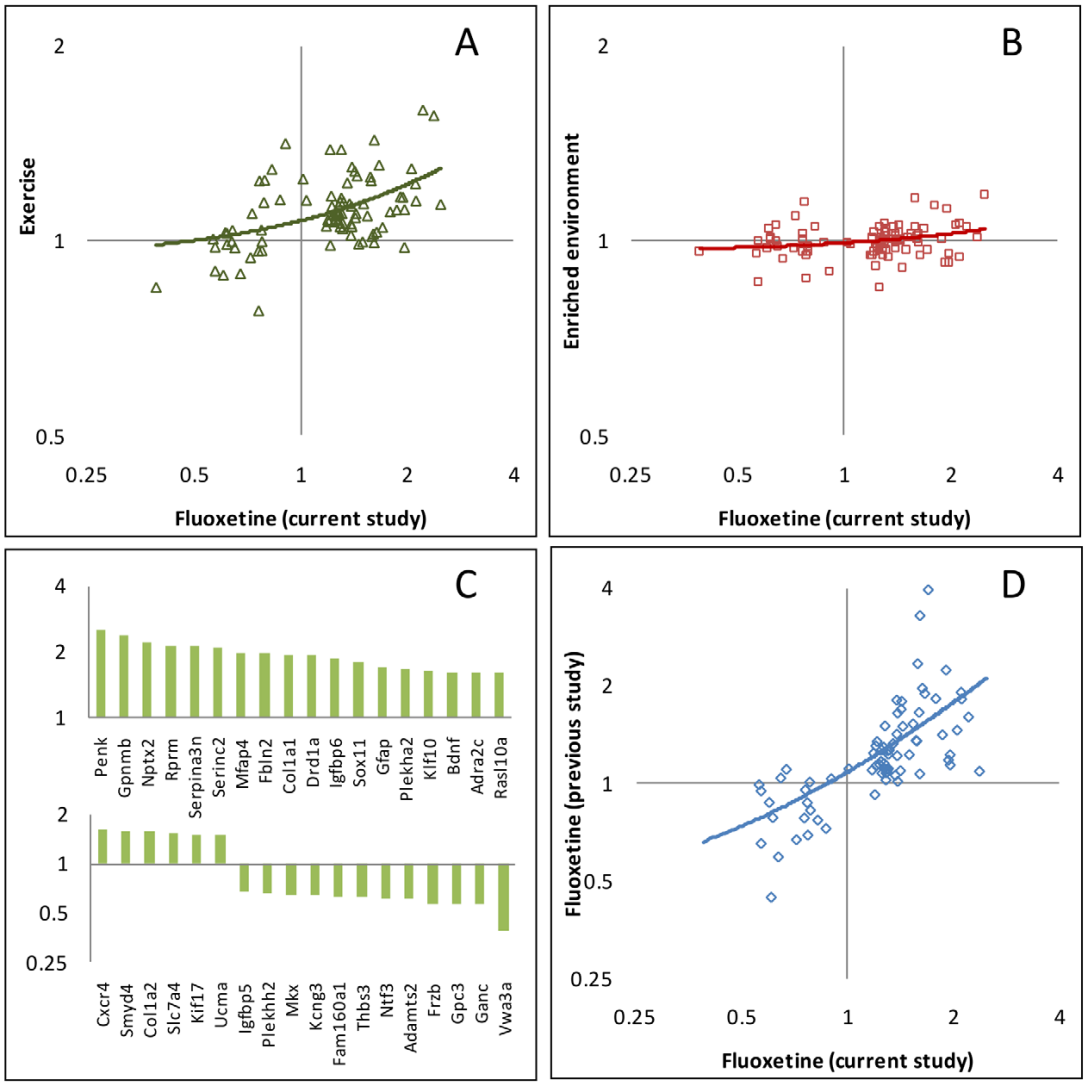
Hippocampal gene expression profiles in response to different antidepressant treatments. (A) Changes in expression following exercise compared with the changes in the fluoxetine treated group. Each point is the average expression level of the treated group divided by the average expression level in the control group for one of the 87 genes showing significant differential expression. The Y and X axes are on a log scale, base 2. The line is the best fit linear regression. (B) Changes in expression in an enriched environment as a function of the changes in the fluoxetine treated group. (C) Changes in expression for 36 genes (out of 87) with the highest fold change following chronic fluoxetine treatment compared to controls. (D) Comparison of the effect of fluoxetine on gene expression in the current study with the results obtained by Miller et al. (2008) [17].

Similar to the results obtained for neurogenesis, the gene expression data pointed to a closer relationship between voluntary exercise and the drug treatment than between enriched environment and either of the other interventions (Figure 4 A and B). The correlation between the expression ratios (treatment vs. control) of the two treatment groups, fluoxetine and voluntary exercise, across all genes was 0.49, relative to a correlation of 0.15 between the enriched environment and fluoxetine.

Out of the 87 genes showing significant differential expression between the groups, four genes had a *P*<0.05 in the enriched environment group, 37 had a *P*<0.05 in the exercise group, and 84 had a *P*<0.05 in the fluoxetine group, in comparisons with the control group. Surprisingly, out of the 87 genes, the expression of 34 genes was significantly (*P*<0.05) altered in both the fluoxetine and the exercise groups, and in 30 cases the change was in the same direction (upregulated or downregulated) in both groups. Moreover, even the genes that were not significant (*P*>0.05) in the exercise group showed the same trend as was observed in the fluoxetine group. Of the 84 genes showing significant differential expression in the fluoxetine group (Figure 4C), 60 were upregulated, and 24 were downregulated following fluoxetine treatment. Out of the 60 fluoxetine upregulated genes, 55 also showed higher expression in the exercise group relative to controls, and out of the 24 fluoxetine downregulated genes, 13 were also lower in the voluntary exercise group (*P* = 0.00027) (Table S1).

**Figure 5:**
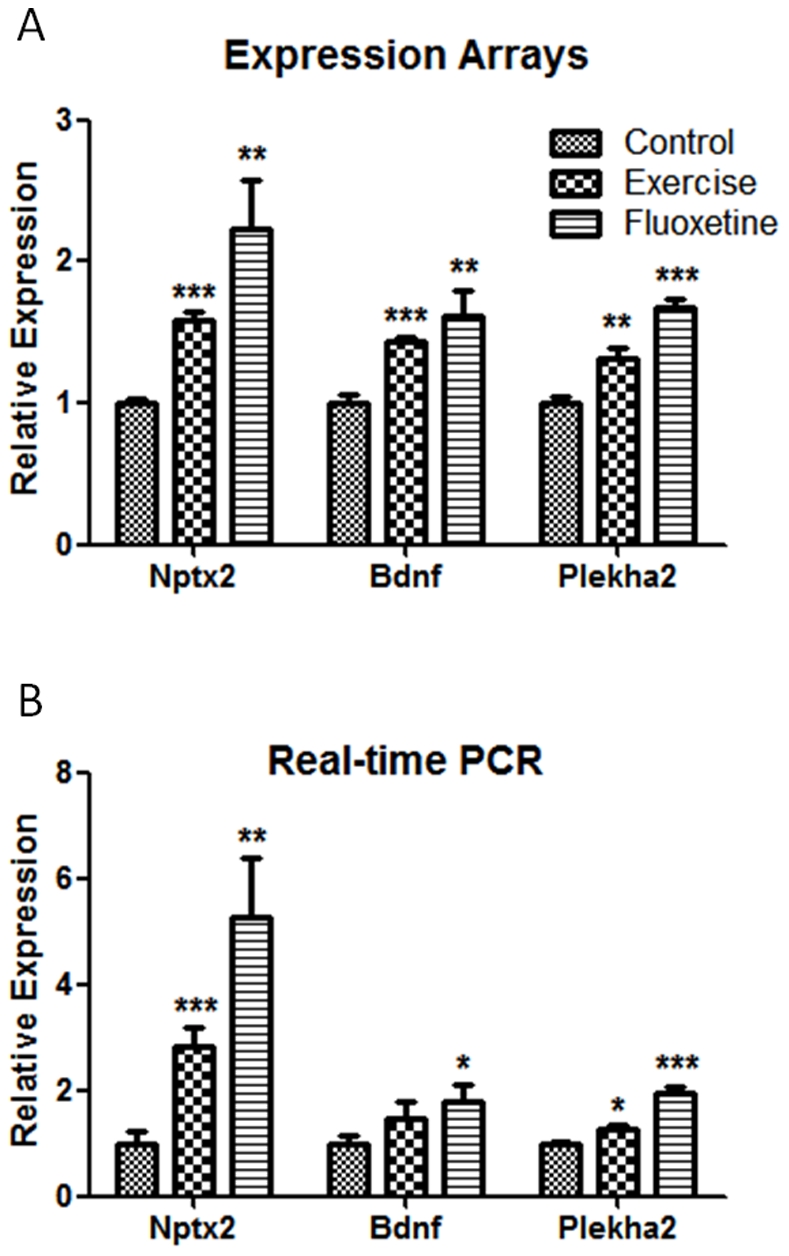
Validation by real-time PCR of three genes that show differential expression in the microarray analysis. The expression of Nptx2, Bdnf and Plekha2 is shown for the fluoxetine and exercise groups relative to control. (A) Relative expression based on (A) expression arrays and (B) real-time PCR. Values are means ± SEM. *P<0.05, **P<0.01, ***P<0.001.

Among the genes upregulated in both fluoxetine and voluntary exercise groups was Bdnf, a gene extensively studied as being a possible mediator of the effect of antidepressant treatments [16]. We further measured the expression of three differentially expressed genes (Bdnf, Nptx2 and Plekha2) by real-time PCR (Figure 5). Similar to the array results (Figure 5A) the three genes were upregulated relative to control (Figure 5B), with highest expression in the fluoxetine group.

In order to further exclude the possibility of an artefactual explanation for our results we searched for expression data carried out by others on mice treated with any of the antidepressant treatments, fluoxetine or voluntary exercise. We found one study in the Gene Expression Omnibus (GEO), which profiled hippocampal gene expression from DBA/2J males chronically treated with fluoxetine [17]. Of the 87 genes regulated by antidepressant treatment in our study, there were data for 75 on the arrays of Miller et al. [17]. Of the 56 hippocampal fluoxetine upregulated genes in our study, 55 had a ratio above 1 in the data of Miller et al. (mean ratio = 1.45) and out of the 19 down regulated genes, 15 had a ratio below 1 (mean ratio = 0.83) in the previous study (*P* = 1.28×10^−11^). The correlation between the expression ratios in the two studies (for the 75 significant genes) was 0.57, with corresponding *P* = 1.27×10^−7^ (Figure 4D). This very strong correlation confirms that the expression results we obtained are unlikely to be due to factors specific to our experiment.

We also compared our results with data from a study that examined gene expression in mice treated with antipsychotic drugs (unpublished data, GEO: GSE6511). We analyzed the expression from brains of mice treated with Clozapine, Haloperidol and Control (n = 3 in each group) for four weeks. Of the 87 genes differentially expressed in our data, 48 were available in this dataset, and only 1 of them (Rgs8) was among the 100 most significantly differentially expressed genes following chronic treatment with either Clozapine or Haloperidol. We conclude that the genes influenced by antipsychotic drugs do not overlap with the genes influenced by fluoxetine or exercise.

### A gene co-expression network analysis identifies modules associated with antidepressant interventions

**Figure 6:**
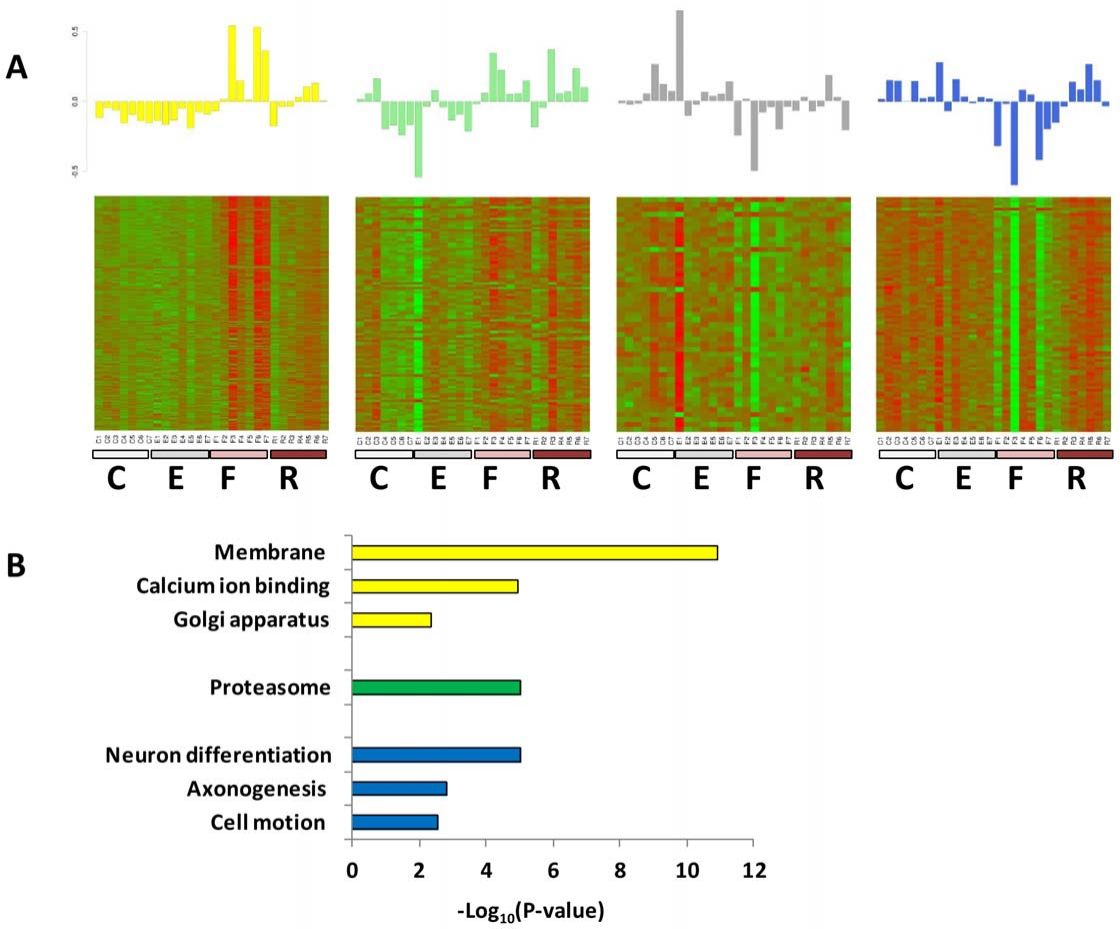
Modules significantly correlated with exercise and fluoxetine. (A) (Upper) Barplots of the values of the module eigengene (i.e., the first principal component) derived from singular value decomposition are displayed for the four modules significantly correlated with exercise and fluoxetine (yellow, light green, dark grey and royal blue). (Lower) Heat maps depicting expression levels for each module (Red, increased expression; green, decreased expression relative to the mean), for all genes (rows) and group of mice (columns): C, control; E, enriched environment; F, fluoxetine; R, exercise (running wheel). (B) Gene enrichment and functional annotation analyses of genes in the different modules using DAVID bioinformatics [14], [15]. No significant enrichment was found in the Dark grey module.

To study the relation between antidepressant action and gene expression, we applied a weighted-gene co-expression network analysis (WGCNA) to identify modules of co-expressed genes. A network was constructed using a pairwise correlation matrix of the expression of the 4,000 most variable genes across all samples. We performed hierarchical clustering on the topological overlap dissimilarity matrix and found 11 modules after merging those that were highly correlated. In order to discover modules associated with the antidepressant treatments, we tested the correlation between each module's eigengenes (defined as the first principal components) and both interventions (fluoxetine and exercise versus control). Four modules showed significant (*P*<0.05) association with antidepressant treatments (Table 1, and Figure 6A). For each gene within the modules we also calculated the gene significance – the correlation between the expression profile across samples of each gene and the antidepressant treatment. In addition, for each module, and for each gene, we calculated the module membership, which is the correlation between the gene expression and the module eigengene (summarized in Table 1). The correlation between the gene significance and the module membership score was significant (*P*<0.05) in the four modules, illustrating that genes significantly associated with antidepressant treatments are often also the most important elements in this modules.

**Table 1:** Modules of co-expressed genes associated with antidepressant interventions.

Module	Yellow	Light green	Dark grey	Royal blue
No. of genes:	310	102	47	87
Correlation^1^:	0.6	0.56	−0.47	−0.37
Significance:	8×10^−4^	0.002	0.01	0.05

	10 most significant genes within each module			
1	**Dpy19l3**	**Snx25**	Leng8	Vwa3a
2	Plekha2	Akirin2	Mfsd5	Igfbp5
3	Kcnk1	Uxs1	R3hdm1	Mkl2
4	Darc	Psmb6	Tnrc6b	Hap1
5	Slc7a14	Tmem223	Psmd2	Pnck
6	Grasp	Park2	Abcb1a	Smarca2
7	Dbc1	Nsg1	Gnb2l1	Ntng1
8	Bdnf	AB041803	N28178	Sorbs1
9	Rgs8	Slc38a2	Pnet-ps	**Rasgrf2**
10	Kif17	Dctn3	**Nktr**	Tub

1 correlation between module eigengene and antidepressant interventions (exercise and fluoxetine).

In bold are the gene with the highest module membership score.

Out of the four modules, two were positively and two negatively correlated with fluoxetine and exercise (Table 1). Gene enrichment and functional annotation analyses revealed that the modules are enriched for specific processes (Figure 6B). The module showing the most consistent effect (Light green) in both fluoxetine and exercise was upregulated compared to control, and was enriched for genes belonging to the proteasome. Another module that was modestly and negatively correlated with fluoxetine and exercise when tested together, was the one most significantly correlated to fluoxetine when tested alone (correlation = −0.61, *P* = 5×10^−4^). This module is downregulated by antidepressants and is enriched for genes involved in neuronal differentiation and maturation (Figure 6B).

### Exercise affects behavior in a similar way to chronic fluoxetine treatment

**Figure 7:**
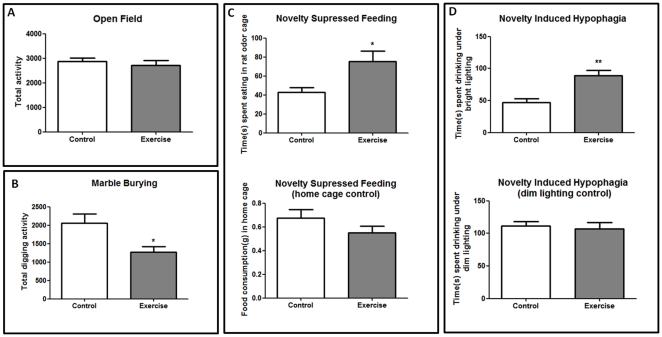
Antidepressant-like behavior in mice engaged in voluntary exercise for 28 days (n = 8). A series of antidepressant-like behavioral tests were conducted. (A) There was no significant difference in the activity in the open field test. (B) Exercise group showed lower levels of digging activity in a new cage with a 5 cm layer of sawdust bedding. (C) In the novelty suppressed feeding test, the exercise group increased the time spent in eating in a rat odor cage compared to controls. No significant differences between the groups in food consumption at the home cage. (D) In the novelty induced hypophagia test, the exercise group spent more time drinking sweet milk under bright lighting cage condition, but no significant differences were observed under dim lighting cage conditions. Values are means ± SEM. *P<0.05, **P<0.01.

We tested whether voluntary exercise had the expected antidepressant effects by comparing exercising animals to controls in behaviors that were found to be modulated in response to fluoxetine: novelty suppressed feeding [18], marble burying and digging activity in a new environment [19] [20], and novelty induced hypophagia [21]. Mice given free access to a running wheel did not show significant differences of total activity in an open field arena (Figure 7A). However, they did exhibit significantly less burying and digging activities in a new cage compared to controls (t = 2.69, df = 14, *P*<0.05) (Figure 7B). In the novelty suppressed feeding test, the exercise group showed a significant increase in the time spent in eating food in an aversive environment after overnight food deprivation (t = 2.72, df = 14, *P*<0.05) (Figure 7C). However, there was no difference in the amount of food consumption in the home cage within 30 minutes immediately after the novelty suppressed feeding test (*P* = 0.18, t = 1.38) (Figure 7D). Similarly, in the novelty induced hypophagia test, the exercise group showed a significant increase in the time spent drinking in a bright cage (*P*<0.01, t = 4.1) (Figure 7E), with no differences in a dark cage (*P* = 0.7, t = 0.38) (Figure 7F).These results confirm that voluntary exercise affects behavior in way which is comparable to chronic fluoxetine treatment.

## Discussion

Using a neurogenomics approach to uncover the changes following antidepressant treatments at the molecular, neuronal and behavior levels, we found significant alterations in multiple levels that are common to both exercise and fluoxetine treatments, but are not shared with environmental enrichment. Our results suggest that there is a shared mechanism underlying the antidepressant effect of fluoxetine and exercise. We have identified similar changes in neurogenesis and structural plasticity in the hippocampus of mice following chronic fluoxetine treatment and voluntary exercise. A co-expression network analysis revealed changes of specific groups of genes in the mouse hippocampus in response to antidepressant treatments.

Gene expression is subject to a large number of confounds, unrelated to the intervention under examination, but we believe our findings are robust for a number of reasons. Most strikingly, an independent analysis identified a highly significant overlap in the differentially expressed set of genes: the correlation between the expression ratios in the two studies was 0.57, with a corresponding of *P* = 1.27×10^−7^. Second, the variances in our gene expression data were low, due to the fact that we used an exon array which has approximately 40 probes per gene, providing a very accurate measurement of gene expression. Third, among the genes upregulated in both the fluoxetine and exercise groups was Bdnf, a gene extensively studied as being a possible mediator of the effect of antidepressant treatments [16].

Several consistent changes were observed in response to exercise and fluoxetine treatments, which may reveal a core mechanism of antidepressant action. Increase in neurogenesis was observed in both treatments as indicated by staining of immature neurons with DCX. Neuronal survival rates, as indicated by BrdU+/NeuN+ double labeling, were also higher in the exercise and fluoxetine groups. In addition, in both groups there was an increase in spine density. These changes at the cellular levels can be also related to the corresponding changes in gene expression. The most consistent finding at the network level was the upregulation of the light green module, which is enriched for genes involved in the function of the proteasome. The upregulation of proteasome genes might be connected to the increase in dendritic spine density, since this process has very high and dynamic degradative demands [22].

Our analyses indicate that genes showing altered expression following antidepressant treatment cluster into specific co-expression networks. These modules allowed us to identify key genes in the pathways affected by antidepressant treatment based on their membership in modules with known biological roles, and to relate the modules to neuronal changes. From these results, we conclude that at least part of the antidepressant effect of exercise and fluoxetine is shared, in particular changes in neurogenesis and dendritic spine density, and that these processes may be further studied to identify new targets for the treatment of depression.

## Supporting Information

Table S1 A list of 87 differentially expressed genes with a q-value<0.05.
(DOCX)
