# Supplementary material for: Neurogenomic Evidence for a Shared Mechanism of the Antidepressant Effects of Exercise and Chronic Fluoxetine in Mice
Source: PLoS One. 2012 Apr 25;7(4):e35901. doi: 10.1371/journal.pone.0035901 (PMC3338479; doi:10.1371/journal.pone.0035901)
Supplement: Table S1 — A list of 87 differentially expressed genes with a q-value<0.05. (DOCX) [file pone.0035901.s001.docx]

TABLE S1

| Gene Symbol | mRNA Accession | transcript cluster id | q-value | Fold change^1^ | | |
| --- | --- | --- | --- | --- | --- | --- |
|  |  |  |  | E | F | R |
| Rasl10a | NM_145216 | 6778426 | 1.5E-05 | 1.02 | 1.61 | 1.02 |
| Kif17 | NM_010623 | 6917979 | 5.7E-05 | 1.01 | 1.51 | 1.14 |
| Col1a1 | NM_007742 | 6783685 | 5.7E-05 | 1.12 | 1.95 | 1.17 |
| Darc | NM_010045 | 6764209 | 5.8E-05 | 0.99 | 1.39 | 1.13 |
| Gfap | NM_010277 | 6791641 | 9.7E-05 | 0.97 | 1.71 | 1.05 |
| Rprm | NM_023396 | 6886809 | 2.7E-04 | 0.94 | 2.13 | 1.15 |
| Serinc2 | NM_172702 | 6925654 | 2.7E-04 | 1.06 | 2.06 | 1.29 |
| Gpnmb | NM_053110 | 6946055 | 4.1E-04 | 1.01 | 2.39 | 1.56 |
| Plekha2 | NM_031257 | 6981113 | 6.5E-04 | 1.05 | 1.67 | 1.31 |
| Mfap4 | NM_029568 | 6781515 | 8.4E-04 | 0.93 | 1.97 | 1.12 |
| Dpy19l3 | NM_178704 | 6966498 | 1.1E-03 | 1.00 | 1.36 | 1.23 |
| Vwa3a | NM_177697 | 6963955 | 2.0E-03 | 0.96 | 0.39 | 0.85 |
| Igfbp6 | NM_008344 | 6833393 | 2.6E-03 | 1.00 | 1.88 | 1.12 |
| Slc7a4 | NM_144852 | 6844283 | 4.4E-03 | 1.03 | 1.55 | 1.09 |
| Slc7a14 | NM_172861 | 6903983 | 4.4E-03 | 1.03 | 1.30 | 1.15 |
| Rasd1 | NM_009026 | 6788723 | 5.2E-03 | 1.05 | 0.83 | 1.29 |
| Kcng3 | NM_153512 | 6857763 | 5.2E-03 | 1.00 | 0.64 | 1.00 |
| Nptx2 | NM_016789 | 6935451 | 5.7E-03 | 1.05 | 2.23 | 1.59 |
| Ganc /// Capn3 | NM_172672 | 6880577 | 5.7E-03 | 0.96 | 0.56 | 1.01 |
| Drd1a | NM_010076 | 6813284 | 6.3E-03 | 0.93 | 1.92 | 1.17 |
| Serpina3n | NM_009252 | 6797579 | 8.0E-03 | 1.06 | 2.12 | 1.22 |
| Dusp4 | NM_176933 | 6975335 | 8.3E-03 | 1.03 | 1.21 | 1.38 |
| Slc26a10 | NM_177615 | 6777915 | 1.1E-02 | 1.10 | 0.73 | 1.10 |
| Homer1 | NM_152134 | 6808997 | 1.2E-02 | 0.99 | 1.02 | 1.25 |
| Rgs2 | NM_009061 | 6762784 | 1.2E-02 | 1.00 | 1.44 | 1.10 |
| Bok | NM_016778 | 6751709 | 1.3E-02 | 1.02 | 1.30 | 1.38 |
| Rgs8 | NM_026380 | 6754137 | 1.3E-02 | 0.95 | 1.32 | 1.15 |
| Grasp | NM_019518 | 6833308 | 1.3E-02 | 1.01 | 1.45 | 1.26 |
| Acvr1 | NM_007394 | 6886957 | 1.3E-02 | 1.03 | 1.39 | 1.06 |
| Aqp4 | NM_009700 | 6863605 | 1.4E-02 | 1.01 | 1.39 | 1.02 |
| Itpr1 | NM_010585 | 6948906 | 1.4E-02 | 0.96 | 0.76 | 0.96 |
| Sox11 | NM_009234 | 6799578 | 1.4E-02 | 1.14 | 1.80 | 1.11 |
| Nrn1 | NM_153529 | 6812375 | 1.6E-02 | 1.01 | 1.28 | 1.10 |
| Svop | NM_026805 | 6941189 | 1.6E-02 | 0.97 | 1.25 | 1.09 |
| Ucma | BC115609 | 6874639 | 1.6E-02 | 0.97 | 1.50 | 1.00 |
| Penk | NM_001002927 | 6919320 | 1.6E-02 | 1.18 | 2.50 | 1.14 |
| Homer3 | NM_011984 | 6976987 | 1.8E-02 | 1.05 | 1.38 | 1.14 |
| Refbp2 | NM_019484 | 6755184 | 1.8E-02 | 0.95 | 0.78 | 1.00 |
| Cxcr4 | NM_009911 | 6762024 | 1.8E-02 | 1.00 | 1.59 | 1.01 |
| 2610019F03Rik | NM_173744 | 6980606 | 1.8E-02 | 1.05 | 1.31 | 1.10 |
| Slc20a1 | NM_015747 | 6881123 | 1.9E-02 | 1.02 | 1.32 | 1.06 |
| Ncan | NM_007789 | 6983162 | 2.0E-02 | 0.97 | 1.28 | 1.07 |
| Kcnk1 | NM_008430 | 6979914 | 2.0E-02 | 0.99 | 1.22 | 1.17 |
| Fam160a1 | NM_172682 | 6906564 | 2.0E-02 | 1.00 | 0.62 | 1.03 |
| Ap1g2 | NM_007455 | 6824799 | 2.0E-02 | 1.15 | 0.77 | 1.04 |
| Plekhh2 | NM_177606 | 6852750 | 2.0E-02 | 0.98 | 0.65 | 0.97 |
| Sel1l3 | NM_172710 | 6938217 | 2.1E-02 | 0.98 | 1.24 | 1.07 |
| Ubash3b | NM_176860 | 6994887 | 2.1E-02 | 0.92 | 1.23 | 1.13 |
| Cort | NM_007745 | 6926933 | 2.1E-02 | 1.03 | 0.76 | 1.24 |
| Klf10 | NM_013692 | 6835089 | 2.1E-02 | 0.95 | 1.64 | 1.03 |
| Mkx | NM_177595 | 6863210 | 2.1E-02 | 1.06 | 0.64 | 1.04 |
| Tspan5 | NM_019571 | 6901780 | 2.5E-02 | 0.95 | 1.19 | 1.08 |
| Cxxc5 | NM_133687 | 6860068 | 2.6E-02 | 0.96 | 1.21 | 0.98 |
| St6galnac4 | NM_011373 | 6876209 | 2.6E-02 | 1.01 | 1.28 | 1.13 |
| Col1a2 | NM_007743 | 6943818 | 2.7E-02 | 1.16 | 1.58 | 1.25 |
| Egr1 | NM_007913 | 6859972 | 2.8E-02 | 0.90 | 0.90 | 1.41 |
| H3f3a /// Gm12657 | XM_907340 | 6847834 | 3.1E-02 | 1.01 | 0.76 | 0.78 |
| Frzb | NM_011356 | 6888151 | 3.3E-02 | 1.00 | 0.57 | 0.98 |
| Serpinb8 | NM_011459 | 6752165 | 3.3E-02 | 1.02 | 1.40 | 1.30 |
| C1ql2 | NM_207233 | 6752529 | 3.5E-02 | 0.97 | 1.40 | 1.05 |
| Adra2c | NM_007418 | 6929881 | 3.5E-02 | 0.99 | 1.62 | 0.99 |
| Smyd4 | NM_177009 | 6782430 | 3.5E-02 | 1.05 | 1.59 | 1.24 |
| 1700054N08Rik | NM_028536 | 6986030 | 3.6E-02 | 0.95 | 1.18 | 1.05 |
| Agrn | NM_021604 | 6927362 | 3.7E-02 | 1.07 | 1.29 | 1.05 |
| C4b /// C4a | NM_011413 | 6855051 | 3.9E-02 | 0.91 | 1.46 | 0.99 |
| Ppm1h | NM_176919 | 6771334 | 3.9E-02 | 0.95 | 1.29 | 1.10 |
| Shmt1 | NM_009171 | 6788791 | 3.9E-02 | 0.96 | 0.79 | 1.15 |
| Igfbp5 | NM_010518 | 6759664 | 3.9E-02 | 0.94 | 0.67 | 0.89 |
| Bdnf | NM_001048139 | 6879925 | 3.9E-02 | 1.02 | 1.62 | 1.43 |
| Klk8 | NM_008940 | 6960178 | 3.9E-02 | 1.06 | 1.42 | 1.28 |
| Thbs3 | NM_013691 | 6899217 | 4.2E-02 | 1.02 | 0.62 | 0.99 |
| Nt5dc3 | NM_175331 | 6769559 | 4.2E-02 | 0.98 | 1.24 | 1.10 |
| St3gal5 | NM_011375 | 6946954 | 4.2E-02 | 1.01 | 1.20 | 1.07 |
| Lpin2 | NM_022882 | 6852034 | 4.3E-02 | 0.97 | 1.32 | 1.04 |
| Mthfd1l | NM_172308 | 6771920 | 4.4E-02 | 0.98 | 0.79 | 1.24 |
| Pcdh20 | NM_178685 | 6826611 | 4.4E-02 | 0.99 | 0.87 | 1.16 |
| Adamts2 | NM_175643 | 6780811 | 4.4E-02 | 0.98 | 0.60 | 0.88 |
| Gpc3 | NM_016697 | 7016826 | 4.4E-02 | 0.86 | 0.57 | 0.90 |
| Fbln2 | NM_001081437 | 6947987 | 4.4E-02 | 0.96 | 1.97 | 0.98 |
| Slc35f3 | NM_175434 | 6979919 | 4.4E-02 | 0.99 | 1.30 | 1.16 |
| Ltk | NM_206941 | 6890257 | 4.4E-02 | 0.88 | 0.78 | 0.97 |
| Ntf3 | NM_008742 | 6957217 | 4.4E-02 | 1.05 | 0.61 | 1.03 |
| Spns2 | BC025823 | 6789541 | 4.4E-02 | 0.99 | 1.04 | 1.15 |
| Inha | NM_010564 | 6750639 | 4.4E-02 | 0.97 | 0.72 | 0.94 |
| Paqr9 | NM_198414 | 6991531 | 4.5E-02 | 1.05 | 1.26 | 1.08 |
| Thbs2 | NM_011581 | 6853899 | 4.5E-02 | 0.85 | 1.26 | 1.11 |
| Matn2 | NM_016762 | 6829659 | 4.7E-02 | 1.03 | 1.43 | 1.09 |

1 The fold change was calculated between mean values of control and antidepressant interventions.

E, enriched environment; F, fluoxetine; R, voluntary running
